# Psychological Health and Social Wellbeing Among Older Adults

**DOI:** 10.3390/healthcare14010065

**Published:** 2025-12-26

**Authors:** Doreen W. H. Au

**Affiliations:** School of Nursing and Health Sciences, Hong Kong Metropolitan University, Kowloon, Hong Kong; dau@hkmu.edu.hk

## 1. Introduction

In contemporary health discourse, the terms “mental health,” “psychological health,” and “social wellbeing” are prominent, collectively representing the emotional, cognitive, and social facets of overall wellness. While good mental health is described as a state enabling individuals to manage stress, cultivate abilities, and contribute meaningfully to their communities [1], psychological health and social wellbeing extend this paradigm by encapsulating resilience and positive functioning—vital components for navigating life’s complexities, fostering and maintaining meaningful relationships, building social networks, and nurturing a sense of belonging [2]. The interplay among these concepts holds particular significance for older adults, as life transitions and health challenges often put a strain on their support systems.

The global increase in the aging population presents both significant challenges and unique opportunities to elevate the psychological health and social wellbeing of older adults. As this demographic continues to expand, there is a pressing need for effective strategies that address the multifaceted factors influencing their overall quality of life. In this context, this Special Issue (SI) aims to illuminate critical gaps in our understanding and to highlight research that provides insights into the diverse needs of older adults, setting the stage for future exploration in this vital area of study.

The seven studies featured in this SI underscore the need to improve both psychological health and social wellbeing, focusing on issues such as social isolation, suicidal ideation, economic hardships, and digital engagement. These studies highlight the value of adopting targeted, integrated strategies tailored to the specific circumstances of this demographic, while also emphasizing the importance of accounting for gender differences in addressing the complexities of aging.

## 2. Overview of Contributions

A prominent theme across the studies presented in this SI is the essential role of social connections in fostering psychological wellbeing. The evidence suggests that regular engagement with family and friends significantly improves perceptions of care and mitigates feelings of isolation (Contributions 1–3). Therefore, cultivating social engagement should be a cornerstone of efforts aimed at improving the psychological wellbeing of older adults.

Equally important is the influence of socioeconomic factors on mental health outcomes. Research shows a clear correlation between lower socioeconomic status and negative psychological effects, particularly among marginalized groups such as informal waste pickers and older individuals facing economic hardships (Contributions 4 and 5). This connection highlights an urgent need for policies that consider financial stability and social support as integral components of mental health care for older adults.

Depression is a consistent theme among the presented studies, exerting a profound impact on cognitive function, social engagement, and suicidal ideation (Contributions 1 and 6). The findings of these studies prompt critical examination of the strategies through which mental health interventions can be optimized to address depression among vulnerable populations, particularly those living alone or in precarious employment situations. Structured programs like the Awareness, Courage, and Love initiative aim to combat loneliness and enhance psychological wellbeing (Contribution 2). These proactive approaches should be integrated into community frameworks to build resilience and foster a sense of belonging among older adults.

Some of the presented studies provide unique insights that deepen our comprehension of these issues. For example, Lorber et al. emphasize person-centered care, advocating for customized approaches that acknowledge each older adult’s individuality, thereby enhancing their overall satisfaction and psychological health (Contribution 3). Similarly, Morais et al. focus on the validation of a mindfulness scale, investigating its effectiveness in alleviating occupational stress among older farmers (Contribution 5). Additionally, the notable disparities experienced by informal waste pickers in Hong Kong draw attention to the essential role that gender and socioeconomic factors play in shaping mental health within marginalized communities (Contribution 4). Collectively, these findings underscore the critical need for targeted interventions to improve psychological health, especially among vulnerable groups that frequently do not receive adequate attention from society.

The nuanced examination of digital engagement by Jeon et al. distinguishes between the purposes of internet use, demonstrating that while online activity can enhance health outcomes, the context and nature of this engagement play a critical role in its effectiveness (Contribution 7). This insight highlights a crucial area for further exploration in an increasingly digital world.

In summary, the intricate interplay between psychological wellbeing, social dynamics, and socioeconomic factors presents multi-faceted challenges and opportunities in mental health care for older adults. Moving forward, it is essential to acknowledge the diversity of experiences within this population. This body of research could inform collaborative efforts to significantly enhance the health and wellbeing of older adults.

## 3. Future Priorities

Despite considerable progress in this field, significant gaps in the literature remain. There is a pressing need for longitudinal studies that track the trajectories of psychological health and social wellbeing over time, as most research currently relies on cross-sectional findings. Such studies could provide deeper insights into how these aspects evolve throughout the aging process, informing more effective interventions and policies.

While the articles in this SI address various facets of psychological health and social wellbeing, future research should aim to uncover the synergies between these areas, creating comprehensive frameworks for understanding and improving quality of life in older individuals. Collaborative, cross-disciplinary efforts are necessary to develop holistic approaches to the complexities of psychological health and social wellbeing in older adults.

Another notable gap exists in our understanding of environmental impacts on the psychological health and social wellbeing of older adults. Factors such as climate change, urbanization, and access to green spaces can significantly influence mental health outcomes. Research should examine how environmental changes contribute to feelings of isolation, stress, and overall mental health. Understanding these environmental determinants is vital for developing supportive policies that foster healthier and more sustainable living environments for older adults. 

Moreover, there is a need for research on the effectiveness of specific policies and community interventions aimed at enhancing psychological health and social wellbeing. While we recognize the shortcomings in support systems for older adults, evaluating the real-world impacts of such measures is essential for designing effective strategies to benefit this demographic.

As technology plays an increasingly important role in fostering social engagement and wellbeing, further exploration of how various digital platforms can be effectively utilized is necessary. Ensuring that these technologies are accessible and user-friendly for older adults is another area that requires more in-depth investigation.

## 4. Conclusions

In light of the identified research gaps, there has been a call for a second edition of this SI that prioritizes the aforementioned research areas. Researchers, practitioners, and policymakers are invited to submit innovative studies that tackle the urgent needs and further examine the complex landscape of psychological health and social wellbeing in older adults. Of particular interest is research involving longitudinal analyses; intersectionality concerning age, gender, and socioeconomic status; the environmental impacts on wellbeing; community initiatives; and effective applications of technology to enhance psychological health and social wellbeing.

By collaboratively advancing our knowledge in these areas, we can create a more nuanced and effective approach to enhancing the lives of older adults. The contributions featured in this SI illuminate critical pathways for intervention, but the journey does not end here. Continued efforts should be made to enrich academic discourse and improve policies and community initiatives aimed at fostering a well-being economy that values and supports our aging population.

## References

[1] World Health Organization Mental Health: Key Facts. https://www.who.int/news-room/fact-sheets/detail/mental-health-strengthening-our-response.

[2] Kim E.S., Tkatch R., Martin D., MacLeod S., Sandy L., Yeh C. (2021). Resilient Aging: Psychological Well-Being and Social Well-Being as Targets for the Promotion of Healthy Aging. Gerontol. Geriatr. Med..

